# Reconceptualizing the HIV Epidemiology and Prevention Needs of Female Sex Workers (FSW) in Swaziland

**DOI:** 10.1371/journal.pone.0115465

**Published:** 2014-12-22

**Authors:** Stefan Baral, Sosthenes Ketende, Jessie L. Green, Ping-An Chen, Ashley Grosso, Bhekie Sithole, Cebisile Ntshangase, Eileen Yam, Deanna Kerrigan, Caitlin E. Kennedy, Darrin Adams

**Affiliations:** 1 Key Populations Program, Center for Public Health and Human Rights, Department of Epidemiology, Johns Hopkins School of Public Health, Baltimore, Maryland, United States of America; 2 Population Services International, Mbanane, Swaziland; 3 Rock Of Hope, Mbabane, Swaziland; 4 Swaziland National AIDS Program, Strategic Information Division, Ministry of Health, Mbabane, Swaziland; 5 Department of Population, Family, and Reproductive Health, Johns Hopkins School of Public Health, Baltimore, Maryland, United States of America; 6 Department of International Health, Johns Hopkins School of Public Health, Baltimore, Maryland, United States of America; 7 Department of Health, Behavior, and Society, Johns Hopkins School of Public Health, Baltimore, Maryland, United States of America; University of the Stellenbosch, South Africa

## Abstract

**Background:**

HIV is hyperendemic in Swaziland with a prevalence of over 25% among those between the ages of 15 and 49 years old. The HIV response in Swaziland has traditionally focused on decreasing HIV acquisition and transmission risks in the general population through interventions such as male circumcision, increasing treatment uptake and adherence, and risk-reduction counseling. There is emerging data from Southern Africa that key populations such as female sex workers (FSW) carry a disproportionate burden of HIV even in generalized epidemics such as Swaziland. The burden of HIV and prevention needs among FSW remains unstudied in Swaziland.

**Methods:**

A respondent-driven-sampling survey was completed between August-October, 2011 of 328 FSW in Swaziland. Each participant completed a structured survey instrument and biological HIV and syphilis testing according to Swazi Guidelines.

**Results:**

Unadjusted HIV prevalence was 70.3% (n = 223/317) among a sample of women predominantly from Swaziland (95.2%, n = 300/316) with a mean age of 21(median 25) which was significantly higher than the general population of women. Approximately one-half of the FSW(53.4%, n = 167/313) had received HIV prevention information related to sex work in the previous year, and about one-in-ten had been part of a previous research project(n = 38/313). Rape was common with nearly 40% (n = 123/314) reporting at least one rape; 17.4% (n = 23/314)reported being raped 6 or more times. Reporting blackmail (34.8%, n = 113/314) and torture(53.2%, n = 173/314) was prevalent.

**Conclusions:**

While Swaziland has a highly generalized HIV epidemic, reconceptualizing the needs of key populations such as FSW suggests that these women represent a distinct population with specific vulnerabilities and a high burden of HIV compared to other women. These women are understudied and underserved resulting in a limited characterization of their HIV prevention, treatment, and care needs and only sparse specific and competent programming. FSW are an important population for further investigation and rapid scale-up of combination HIV prevention including biomedical, behavioral, and structural interventions.

## Background

Human Immunodeficiency Virus (HIV) is hyperendemic in Swaziland with a prevalence of approximately 26.1% in 2007 among reproductive age adults [1]. This equates to an estimated 170,000 adults living with HIV in 2012. In addition, HIV prevalence in Swaziland is higher among women, who represent 59% of the people living with HIV (PLWHIV), an increase since 2001 when 57% of the PLWHIV were women [2]. While the prevalence of HIV has increased, similar to other generalized epidemic settings the incidence rate likely peaked in approximately 1999 at almost 6% [3]. There appears to have been declines in incidence with 6,054 person years (PY) of follow up data from 11,880 HIV-negative individuals enrolled into a longitudinal cohort and followed from December 2010 – June 2011 as part of the Swaziland HIV Incidence Measurement Survey (SHIMS) study [4], [5]. Incidence was nearly twice as high among women as compared to men (3.14/100PY vs. 1.65/100PY) with overall incidence of approximately 2.4% (95% CI 2.1–2.7%). The highest incidence rate of 4.2% was observed among women 20–24 years old, with a second peak of incidence at 4.2% in women 35–39 years old. While sex work was not assessed in the SHIMS study, higher number of partners and self-reported pregnancy were strongly associated with incident HIV infection. In addition, women who reported not being married or living with partner were also at higher risk of HIV acquisition (adjusted Hazard Ratio of 3.1, 95% CI 1.6–5.7) [4].

There have been limited epidemiologic studies evaluating the burden of HIV among FSW in the generalized epidemics of Sub-Saharan Africa (SSA) [6]. However, where data are available, there is a consistent trend of high burdens of HIV among female sex workers (FSW). A meta-analysis of HIV prevalence studies including 21,421 FSW across SSA demonstrated FSW had more than 14 times increased odds of living with HIV compared to other women [7]. Specifically, the HIV prevalence among FSW in SSA was 36.9% compared to 7.4% among reproductive age women. In a study of 1,050 women in Swaziland and Botswana, 5% reported transactional sex, and this was significantly associated with food insecurity [8]. There are also some data highlighting the higher HIV prevalence of women with a greater number of sexual partners, including the most recent Swaziland Demographic Health Survey (DHS) which found that HIV prevalence was 52.3% among women reporting two or more partners in the preceding 12 months as compared to 38.8% in those reporting zero partners [9]. The report did not assess sex work, making it impossible to assess to what extent commercial sex may have been involved in these higher numbers of sexual partners. Small rapid assessments exploring the dynamics and practices of FSW were conducted in Swaziland in 2002 and 2007 [10]. While sample sizes were small, these two studies highlighted a population at high risk for HIV acquisition and transmission. In a qualitative study among 20 FSW living with HIV in Swaziland, Fielding-Miller (2014) describes a cyclical pathway of hunger, food insecurity, social stigma, sex work, and HIV infection{Fielding-Miller, 2014 #62}. A recent study of FSW from Botswana, Namibia, and South Africa also demonstrated high levels of risk for both HIV and human rights abuses among FSW [11]. Specifically, participatory methods were used to identify high levels of sexual and physical abuse, as well as extortion, from law enforcement officers such as police and border guards [11]. The prevalence of these abuses experienced by FSW in Swaziland, where sex work is criminalized, is unknown. A study of sex workers in east and southern Africa found that participants had unmet health needs such as diagnosis and treatment of sexually transmitted infection and access to condoms [12]. Many sex workers in the same study reported they were denied treatment for injuries following physical or sexual assault or faced hostility from health providers.

The 2009 Swaziland Modes of Transmission (MoT) study reported that major drivers of HIV incidence included multiple concurrent partnerships before and during marriage as well as low levels of male circumcision [3]. The MoT suggested that both sex work and male-male sexual behaviors are infrequently reported and are potentially minor drivers of HIV risk in the widespread epidemic of Swaziland [3]. The MoT further highlighted that it is difficult to accurately conclude the role of key populations in larger transmission dynamics as well as their respective vulnerabilities and health care needs because of limited data.

While there has been no targeted HIV prevalence study among FSW in Swaziland, there are several emerging studies exploring concentrated HIV subepidemics in the context of broadly generalized HIV epidemics in other countries in the region [13]. Taken together, these studies suggest that even in a generalized epidemic setting such as Swaziland, these women likely carry a disproportionate burden of HIV due to a confluence of biological, behavioural, and structural risks for HIV infection. This study aimed to describe an unbiased estimate of HIV prevalence among FSW in Swaziland and characterize behavioral factors associated with HIV infection, including individual sexual practices, the composition of sexual networks, concurrent partnerships, substance use, and access to clinical health care and prevention services.

## Methods

Sampling methods have been previously described [14]. Briefly, a respondent-driven-sampling (RDS) survey was completed between August and October, 2011 of 328 adult women who reported selling sex for money in the previous 12 months in Swaziland [15]. Each participant completed a structured survey instrument and biological HIV and syphilis testing and counseling according to Swazi national guidelines. The survey instrument included a comprehensive assessment of HIV risk with modules including demographics, human rights issues, sexual practices, HIV-related knowledge, condom negotiation, social capital, and reproductive history [16]. Interviews lasted approximately one hour and were conducted in private rooms with trained staff and with no personally identifiable information collected at any point.

### Analysis

Population weights were computed separately for each variable by the data-smoothing algorithm using RDS for Stata [17]. The weights were used to estimate RDS-adjusted univariate estimates with bootstrapped 95% confidence intervals. Crude bivariate analysis was also conducted to assess the association of HIV status with demographic variables as well as selection of variables that were either expected or have been shown to be associated with HIV status in the literature [16]. All demographic variables were then included in the multivariate logistic regression model regardless of estimated strength of their crude bivariate association with HIV status. Non-demographic variables were included in the multivariate model if the chi-square p-value of association with HIV status was less than or equal to 0.25.

Although regression analyses of RDS data using sample weights is complicated due to the fact that weights are variable-specific, as noted by Johnston et al., we conducted RDS-adjusted bivariate and multivariate analyses and used the estimates in our sensitivity analyses of the crude estimates [18]. The adjusted odds ratio estimates differed little from the unweighted estimates in both bivariate and multivariate analyses, and therefore we only report the unweighted odds ratios.

The last column in Table 1 shows homophily estimates - a measure of the extent to which participants were likely to recruit participants with a similar characteristic to themselves rather than at random [19]. Homophily values range from −1 to +1. A value of 0 represents random recruitment, negative values represent less likely to recruit from one's own group, and positive values mean more likely to recruit from one's own group. The results show that participants who were 18 to 21 years old, those who have been selling sex for less than two years, and those who grew up in urban areas were slightly more likely to recruit from their own groups than at random. FSW who earn more than fifty percent of their income from selling sex were less likely to recruit from their own group.

**Table 1:** Selected Sociodemographic Characteristics among FSW in Swaziland, 2011.

		n	Crude	RDS-adjusted		Homophily
Variable	Categories (n = observed participants row total)		Percent	Percent	[95% CI]	
Age in years	18–20	62	19.6	33.0	[20.2,45.7]	0.254
	21–25	100	31.5	30.4	[22.4,38.4]	0.144
	26–30	84	26.5	22.8	[13.8,31.7]	0.088
	>31	71	22.4	13.9	[08.3,19.5]	0.133
Number of years selling sex	Under 2	90	28.8	38.3	[27.5,49.1]	0.190
	3–5	98	31.3	32.1	[23.6,40.7]	0.055
	6–10	82	26.2	20.2	[13.2,27.1]	0.087
	11 or more	43	13.7	9.4	[04.4,14.4]	0.001
Education	< = Primary	104	32.8	32.3	[24.1,40.4]	0.021
	> = Secondary	213	67.2	67.7	[59.6,75.8]	0.005
Marital status	Single, never married	278	88.8	90.6	[86.3,94.9]	−0.132
	Married, cohabitate or widowed	35	11.2	9.4	[05.0,37.4]	−0.025
Emp. income other than SW	No	212	66.9	73.2	[66.3,80.0]	−0.136
	Yes	105	33.1	26.8	[19.9,33.7]	0.171
% of total income from SW	< = 50%	26	8.5	6.5	[03.1,09.9]	−0.022
	>50%	280	91.5	93.5	[90.1,96.9]	−0.389
Growing up urban vs. rural	Urban	154	49.0	45.4	[36.4,54.3]	0.234
	Rural	149	47.5	50.4	[41.3,59.5]	0.052
Children	None	77	24.4	25.9	[18.7,33.0]	−0.026
	One	97	30.7	35.6	[27.0,44.2]	−0.085
	Two	81	25.6	24.5	[16.2,32.8]	0.010
	Three or more	61	19.3	14.0	[09.2,18.9]	0.082

All data processing and analyses were conducted using Stata 12.1 [20].

### Missing data

There were only three variables in the final model with missing data: whether a participant experienced symptoms of sexually transmitted infections (STIs) in the last six months (6 missing cases), whether a participant disclosed to a health care worker that she sells sex (1 missing case) and the age at which a participant started selling sex (4 missing cases). Because of the small number of cases with missing data (a combined total of 11 participants), no effort was made to impute the missing data. The eleven cases were excluded in the multivariate regression models.

### Ethical Review

Informed consent was obtained from all participants in either Siswati or English depending on the choice of the participant. Given the anonymous nature of this study, verbal consent was deemed appropriate by the ethical review committees. To note consent, the interviewer initialed the consent statement after verbal consent was obtained. Participants were informed that consent could be withdrawn at any time throughout the study. The study protocol was approved by the National Ethics Committee of Swaziland and the Institutional Review Board of the Johns Hopkins Bloomberg School of Public Health.

## Results

**Figure 1:**
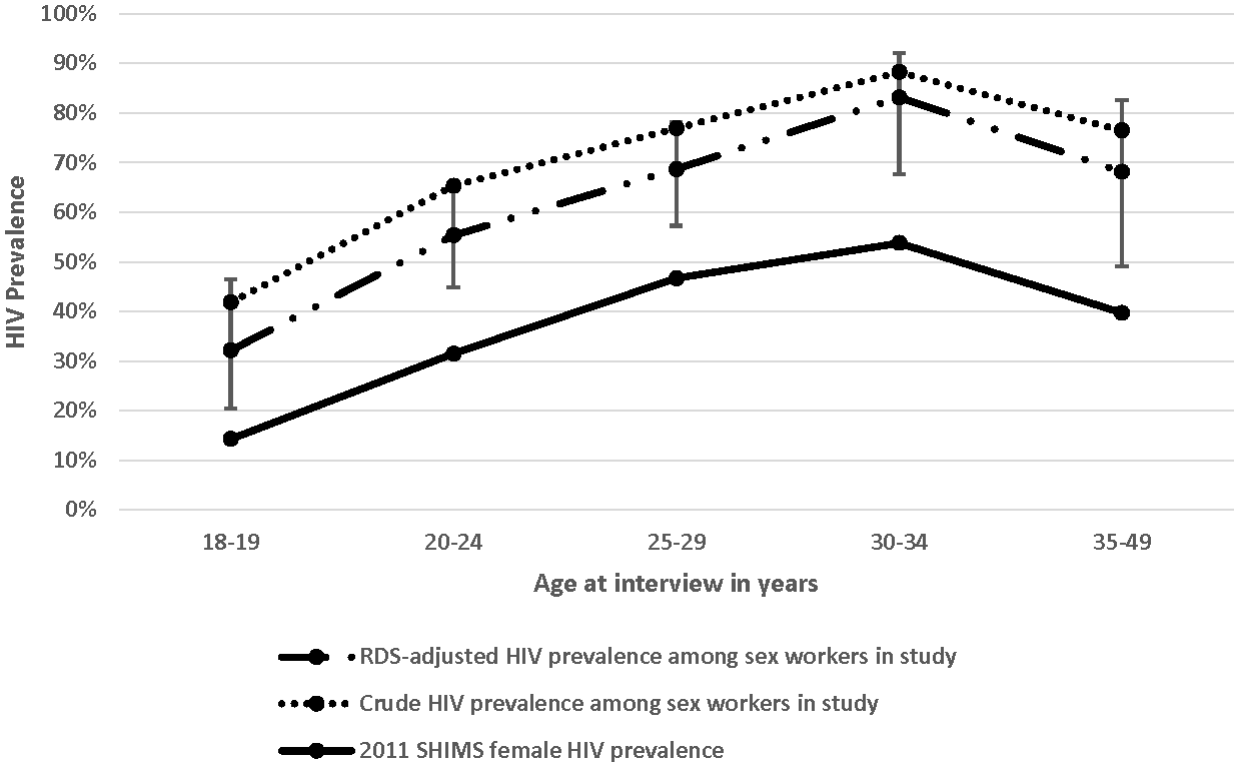
Prevalence of HIV among FSW in Swaziland by age at interview in 2011.

Overall, 223/317 women sampled were living with HIV for a crude prevalence of 70.3% and a RDS-adjusted estimate of 61.0% (95% CI 51.4–70.5%) which is significantly higher than reproductive age women in Swaziland across age categories (Fig. 1). The sample of FSW was predominantly from Swaziland (95.2%, n = 300/316) with a mean age of 21(median 25, results not shown). Approximately three-quarters of the FSW living with HIV were aware of their status, with about 40% receiving either antiviral therapy or co-trimoxazole prophylaxis. Those who were aware of their status as living with HIV did not have fewer partners and were no more likely to wear condoms than those not aware of their status or those who were HIV-negative. Among those who reported they had been diagnosed with HIV, 5.8% tested negative for HIV in this study. The sample was young with over three quarters of the sample being 30 years old or younger (Table 1). The majority of FSW had never been married (88.8%, n = 278/313), over 90% made more than 50% of their total income from sex work (91.5%, 280/306), and more than three-quarters reported having at least one child (75.6%, 239/316).

Just over 40% of the sample had more than 6 clients per week (41.0%, 127/310), with 41% (126/309) reporting more than 7 regular clients per month (Table 2). New clients were common, with 85.4% of the sample (258/302) reporting at least two or more new clients and over a quarter of the sample reporting more than 4 new clients in the last month. Condom use at last sex with both regular and new clients was significantly higher than always wearing condoms with clients in the previous month (p<0.01, data not shown). Nearly 90% of the sample reported at least one non-paying partner within the same time frame with lower condom use than observed in commercial sex (p<0.01, data not shown). More than one quarter (29.1%, 92/316) of the sample reported having been to jail or prison, and slightly more than 5% reported injecting drug use in the preceding 12 months.

**Table 2:** HIV-related risk practices among Female Sex Workers in Swaziland, 2011.

			Crude	RDS-adjusted	
Variable	Categories	n	Percent	Percent	[95% CI]
# of clients per week	1–5	183	59.0	66.5	[57.6,75.4]
	6–10	76	24.5	18.8	[13.3,24.2]
	11 or more	51	16.5	14.7	[08.3,21.2]
Any sex without condom in last six months	No	101	32.0	31.3	[23.7,38.8]
	Yes	215	68.0	68.7	[61.2,76.3]
# of new clients in last 30 Days	One or less	44	14.6	16.4	[09.8,23.0]
	Two	124	41.1	43.4	[33.3,53.5]
	Three	56	18.5	15.2	[09.6,20.9]
	Four	40	13.2	13.1	[07.0,19.2]
	Five	38	12.6	11.8	[06.0,17.6]
Condom at last sex with new client	No	37	12.6	15.2	[07.6,22.8]
	Yes	257	87.4	84.8	[77.2,92.4]
Always condom use with new clients in last 30 days	Not always	137	43.2	43.3	[34.4,52.2]
	Always	180	56.8	56.7	[47.8,65.6]
# of regular clients in last 30 days	0–1	26	8.4	10.0	[01.9,18.1]
	Two	24	7.8	8.5	[03.2,13.8]
	Three	35	11.3	15.9	[09.8,21.9]
	Four	34	11.0	10.0	[04.5,15.6]
	Five	32	10.4	8.1	[03.8,12.3]
	Six	32	10.4	10.7	[05.8,15.5]
	7 plus	126	40.8	36.9	[26.4,47.3]
Condom at last sex with regular client	No	54	17.8	17.1	[09.9,24.2]
	Yes	250	82.2	82.9	[75.8,90.0]
Always condom use with regular clients in last 30 days	Not always	200	63.1	61.4	[52.3,70.4]
	Always	117	36.9	38.6	[29.5,47.7]
# of non-paying partners in last 30 days	None	37	11.7	12.5	[04.8,20.1]
	One	167	53.0	50.8	[42.9,58.7]
	Two	73	23.2	23.6	[16.8,30.3]
	3 plus	38	12.1	13.2	[07.2,19.1]
Condom at last sex with non-paying partners in last 30 days	No	138	51.1	48.9	[39.6,58.2]
	Yes	132	48.9	51.1	[41.8,60.4]
Always condom use with non-paying partners	Not always	247	77.9	79.2	[73.1,85.3]
	Always	70	22.1	20.8	[14.7,26.9]
Injected illicit drugs in last 12 months	No	297	94.3	96.3	[93.7,98.9]
	Yes	18	5.7	3.7	[01.1,06.3]
Non-injected illicit drug use in last 12 months (marijuana, powdered cocaine, and narcotics)	No	212	67.9	78.5	[72.4,84.5]
	Yes	100	32.1	21.5	[15.5,27.5]
Been to jail/prison	No	224	70.9	81.9	[76.5,87.2]
	Yes	92	29.1	18.1	[12.8,23.5]

Knowledge of safe sex practices was limited; only 3 participants knew that anal sex is the highest risk form of sexual transmission, that water-based lubricants are the safest form of lubricant, and that injecting drug use is associated with HIV risk (0.9%, 3/317) (Table 3). While 86% (271/315) reported receiving information about HIV prevention in the last 12 months, approximately half the sample had received HIV prevention information specific to sex work (53.4%, 167/313), and about 10% had ever participated in any research about sex work before (12.1%, 38/313). Over 50% reported peri-anal or peri-genital symptoms consistent with an STI in the last 12 months though less than 15% reported having been diagnosed with an STI in the same timeframe.

**Table 3:** Knowledge of Safe Sex Practices/HIV Risk and Coverage of Prevention Services among FSW in Swaziland, 2011.

			Crude	RDS-adjusted	
Variable	Categories	n	Percent	Percent	[95% CI]
Knowledge that anal sex is highest risk for acquisition	No	278	89.1	90.0	[86.3,92.8]
	Yes	34	10.9	10.0	[7.2,13.7]
Safest types of lubricant to use during sex	Petroleum jelly/Vaseline	51	28.5	30.9	[24.2,38.5]
	Body/fatty creams	10	5.6	2.9	[1.5,5.4]
	Water-based lubricant	38	21.2	17.9	[13.1,23.9]
	Saliva	22	12.3	3.2	[2.1,5.0]
	No lubricant use	58	32.4	45.1	[37.2,53.1]
Knowledge about HIV acquisition risk from injecting illicit drugs	No	12	3.8	4.4	[2.5,7.6]
	Yes	302	96.2	95.6	[92.4,97.5]
All three correct	No	314	99.1	99.9	[99.8,100.0]
	Yes	3	0.9	0.1	[0.0,0.2]
Diagnosed with non-HIV STI in last 12 months	No	259	85.2	91.2	[88.3,93.5]
	Yes	45	14.8	8.8	[6.5,11.7]
Symptoms of genital or anal STI in last 12 months	No	153	49.2	51.9	[46.3,57.4]
	Yes	158	50.8	48.1	[42.6,53.7]
Tested for HIV in last 12 months	No	82	25.9	38.3	[32.5,44.4]
	Yes	234	74.1	61.7	[55.6,67.5]
Given diagnosis of HIV infection in last 12 months	No	140	44.7	55.0	[49.4,60.5]
	Yes	173	55.3	45.0	[39.5,50.6]
Access to condoms when needed	No, difficult, little access	27	8.6	5.3	[3.6,7.6]
	Somewhat difficult access	27	8.6	7.7	[5.3,11.0]
	Somewhat easy access	50	16.0	24.9	[19.7,31.0]
	Very easy access	209	66.8	62.1	[56.2,67.8]
Use of lubricants during sex	No	245	78.0	77.9	[72.9,82.2]
	Yes	69	22.0	22.1	[17.8,27.1]
Access to lubricants when needed	No Access	21	22.6	11.7	[7.4,18.1]
	Difficult or little access	27	29.0	15.8	[10.5,23.1]
	Somewhat difficult access	11	11.8	23.7	[13.9,37.4]
	Somewhat easy access	11	11.8	8.7	[4.7,15.5]
	Very easy access	23	24.7	40.1	[28.8,52.6]
Type of lubricant use during sex	No WBL	290	93.5	96.1	[94.0,97.5]
	Uses WBL	20	6.5	3.9	[2.5,6.0]
Received any information about HIV prevention in last 12 months	No	44	14.0	15.1	[11.4,19.7]
	Yes	271	86.0	84.9	[80.3,88.6]
Received information about HIV prevention for SW in last 12 months	No	146	46.6	61.7	[56.3,66.8]
	Yes	167	53.4	38.3	[33.2,43.7]
Ever participated in research about SW before	No	275	87.9	92.0	[89.1,94.2]
	Yes	38	12.1	8.0	[5.8,10.9]
Ever disclosed SW to any family member	No	220	69.6	75.7	[71.0,79.9]
	Yes	96	30.4	24.3	[20.1,29.0]
Ever disclosed SW to any health care worker	No	234	74.1	86.6	[83.4,89.3]
	Yes	82	25.9	13.4	[10.7,16.6]
Any experienced stigma (12 months)	No	86	27.1	30.1	[25.1,35.6]
	Yes	231	72.9	69.9	[64.4,74.9]
Any perceived stigma (12 months)	No	39	12.3	13.9	[10.3,18.4]
	Yes	278	87.7	86.1	[81.6,89.7]

Human rights abuses were prevalent in this sample. Rape was common with nearly 40% (n = 123/314) reporting at least one rape; 17.4% (n = 23/314) reported being raped 6 or more times. Reporting blackmail (34.8%, n = 113/314) and torture (53.2%, n = 173/314) was also prevalent. Overall, 87.7% (278/317) reported perceived stigma and 72.9% (231/317) reported any experienced event of stigma. Disclosure of sex work to family (30.4%, 96/316) or health care workers (25.9%, 82/316) was reported by only a minority of participants.

**Figure 2:**
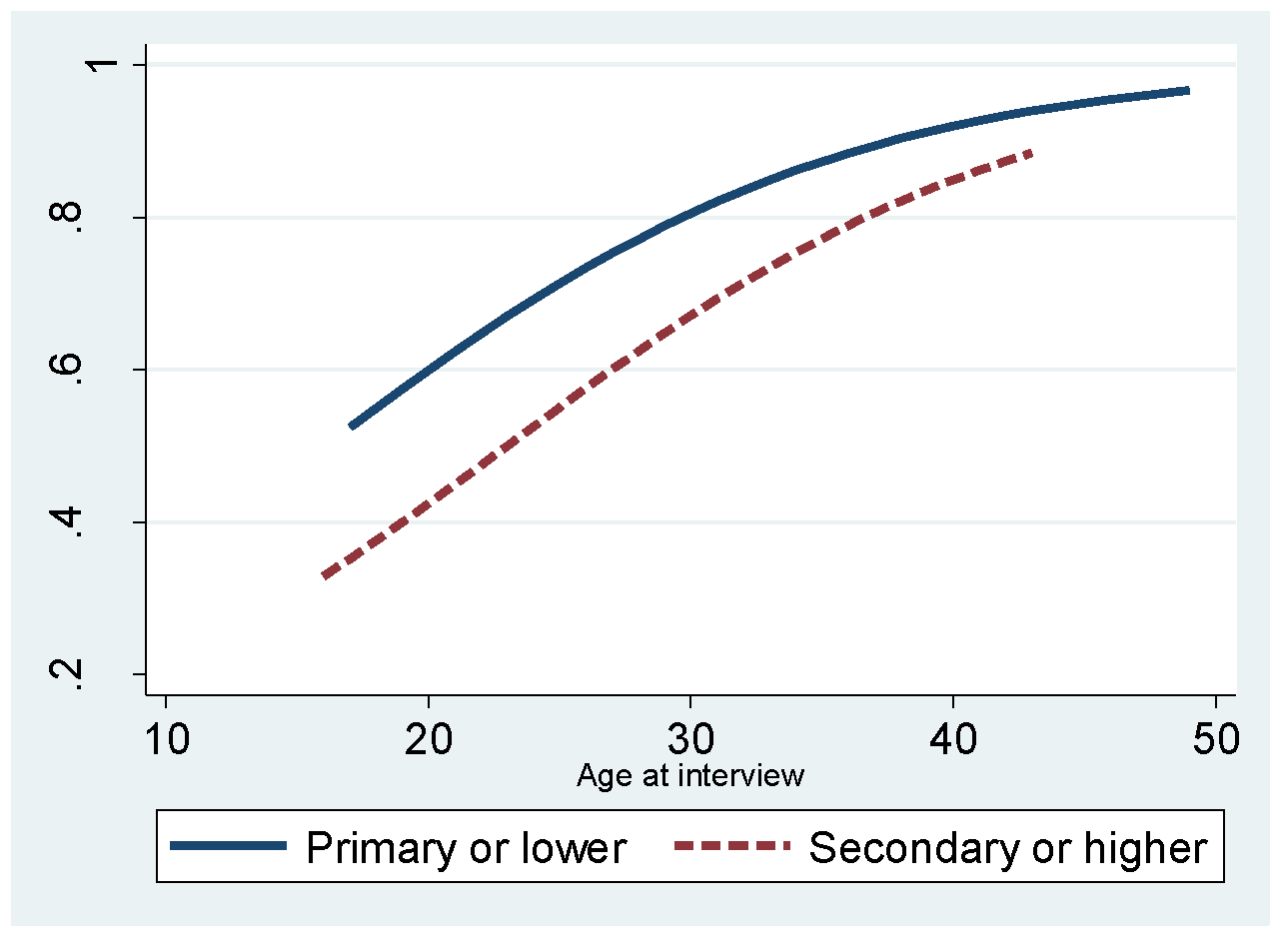
Predicted probabilities of being HIV positive from a crude logistic regression model, by education level, adjusted for age at interview among FSW in Swaziland, 2011.

Statistically significant bivariate associations with HIV included higher age with 83.1% of the 71 women over the age 31 living with HIV (Table 4). In addition, more years selling sex, being less educated, growing up in rural areas, having more children, having been to jail or prison, having had STI symptoms, and ever disclosing sex work involvement to a health care worker were all significantly associated with HIV infection. Significant independent associations found in the multivariate regression model included older age, less education, always wearing condoms with new clients, having had symptoms consistent with an STI in the last year, and disclosing status as a sex worker to a health care worker (Fig. 2, Table 4).

**Table 4:** Significant bivariate and independent associations with HIV serostatus among FSW in Swaziland, 2011.

			HIV test			OR		Adjusted OR^1^	
Variable	Categories (n = observed participants total)	n	% Negative	% Positive	Pvalue	Estimate	[95% CI]	Estimate	[95% CI]
Age in years	20 years or younger	62	54.8	45.2	<0.001	1		1	
	21–25	100	30.0	70.0		2.83**	[1.47,5.47]	2.42*	[1.07,5.47]
	26–30	84	21.4	78.6		4.45***	[2.16,9.17]	3.62**	[1.39,9.43]
	31 and older	71	16.9	83.1		5.97***	[2.69,13.25]	4.40*	[1.38,14.07]
Numbers of years selling sex	Under 2	90	44.4	55.6	<0.001	1		---	
	3–5	98	32.7	67.3		1.65	[0.91,2.98]	---	
	6–10	82	18.3	81.7		3.57***	[1.78,7.18]	---	
	11 or more	43	16.3	83.7		4.11**	[1.66,10.22]	---	
Highest level of education	Primary or lower	104	20.2	79.8	0.010	1		1	
	Secondary or higher	213	34.3	65.7		0.49*	[0.28,0.85]	0.49*	[0.26,0.92]
Marital status	Single never married	278	31.3	68.7	0.084	1			
	Married, cohabitate or widowed	35	17.1	82.9		2.20	[0.88,5.50]	---	
Where did you grow up?	Urban	154	34.4	65.6	0.016	1			
	Rural	149	22.8	77.2		1.77*	[1.07,2.95]		
# of Living Children	None	77	42.9	57.1	0.012	1			
	One	97	28.9	71.1		1.85	[0.98,3.47]	---	
	Two	81	25.9	74.1		2.14*	[1.10,4.19]		
	Three or more	61	18.0	82.0		3.41**	[1.54,7.54]		
Always uses condom with new clients	Not always	137	24.1	75.9	0.058	1		1	
	Always	180	33.9	66.1		0.62	[0.38,1.02]	0.50	[0.27,0.94]
Always uses condom regular clients	Not always	200	30.0	70.0	0.860	1			
	Always	117	29.1	70.9		1.05	[0.63,1.73]	---	
Always uses condom non- paying partner	Not always	247	29.1	70.9	0.713	1			
	Always	70	31.4	68.6		0.90	[0.51,1.59]	---	
Been to Jail or Prison	No	224	34.4	65.6	0.005	1			
	Yes	92	18.5	81.5		2.31**	[1.28,4.19]	---	
Diagnosed with STI in last 12 mts.	No	259	33.2	66.8	0.007	1			
	Yes	45	13.3	86.7		3.23*	[1.32,7.93]	---	
Had STI-symptoms in last 12 mts.	No	153	38.6	61.4		1		1	
	Yes	158	21.5	78.5	0.001	2.29**	[1.39,3.77]	2.80***	[1.56,5.02]
Tested for HIV in last 12 mts.	No	82	40.2	59.8		1			
	Yes	234	25.6	74.4	0.013	1.95*	[1.15,3.32]	---	
Diagnosed with HIV	No	140	58.6	41.4		1			
	Yes	173	5.8	94.2	<0.001	23.04***	[11.2,47.42]		
Received any HIV prevention info in last 12 mts.	No	44	40.9	59.1	0.084	1			
	Yes	271	28.0	72.0		1.78	[0.92,3.43]	—	
Ever disclosed SW to any family member	No	220	30.9	69.1	0.494	1			
	Yes	96	27.1	72.9		1.20	[0.71,2.05]	—	
Ever disclosed SW to any health care worker	No	234	33.3	66.7	0.018	1		1	
	Yes	82	19.5	80.5		2.06*	[1.12,3.80]	2.22*	[1.09,4.50]

Note: Exponentiated coefficients; 95% confidence intervals in brackets.

1 Adjusted for age, education, always using condoms with new clients, STI symptoms in the last 12 months, and ever disclosing sex work to any health care worker.

*p<0.05, **p<0.01, ***p<0.001.

## Discussion

Swaziland has been long recognized as a country with among the highest relative HIV burden in the world [21]. Because there is sustained transmission in the general population with average acquisition and transmission risks, the role of key populations with specific acquisition and transmissions risks such as sex workers has been assumed to be minimal [22]. However, Fig. 1 demonstrates that even in the context of a generalized epidemic, there is a concentrated epidemic among FSW. Few women in this study had any occupation outside of sex work, and even among those who did, the majority of their income was derived from sex work. Thus, these women represent a distinct population from other women who may report occasional transactional sex. While the prevalence of HIV among FSW is high, it is consistent with the few data points available characterizing the burden of HIV in other Southern African countries [7]

The relative contribution or population attributable fraction of sex workers to generalized HIV epidemics is characterized based on two primary components [23], [24]. The first is related to the size of the sexual networks of these women. The second is related to acquisition and transmission risk associated with each sexual act; an outcome of condom usage, type of sexual act, presence of genital or anal ulcerative diseases, and the viral load of the person living with HIV having sex [25]–[28]. Overall, in this sample, sexual networks were large across both commercial and non-paying partners. While periodicity of sexual acts was not measured, assessments of number of partners highlighted that most women had multiple new clients each month, existing or regular clients, and non-paying partners. Further, there were significantly different levels of condom use between partner types with the highest among new clients and lowest among non-paying partners. Always wearing condoms was independently associated with 50% lower odds of living with HIV among FSW, suggesting that in the context of a population with high testing rates and relatively good awareness of HIV-status, the risk may come from new clients where status is unknown. Study participants were less likely to report always wearing condoms as compared to wearing condoms at last sex across partner types, suggesting that always wearing condoms may be a more representative measure of actual condom use. Lastly, even when condoms were used, petroleum-based lubricants were often used which can cause breakdown of the available latex condoms or no lubricant was used, likely associated with the high rates of breakage and slippage of condoms reported by participants (data not shown). While the distribution of condom-compatible lubricants has focused on men who have sex with men, anal sex as a common practice as well as high numbers of partners suggest that this commodity is equally important for FSW.

The burden of HIV in this sample of sex workers is high though no CD4 testing was done to assess eligibility for treatment. Thus, while it is not possible to know the antiretroviral treatment (ART) eligibility of the 60% of FSW in this sample who are not on ART, it is likely that their community viral load was high. Approximately a third of women reported anal sex in the last month in this study with about a tenth of women knowing that this was a high risk act for sexual transmission of HIV (data not shown). A recent review demonstrated that transmission rates in penile-anal sex are approximately 18 times higher than that of penile-vaginal intercourse irrespective of gender [29]. Moreover, reporting symptoms of a peri-anal or peri-genital STI was both common and independently associated with HIV infection in this study. Given large sexual networks, high prevalence of HIV, limited condom usage, and a likely high per-act transmission rate of HIV, the population attributable fraction of HIV among FSW in the widespread epidemic of Swaziland is likely significant.

While most participants were exposed to some form of HIV prevention information in the previous year, most of this was from the media. However, only about half of the women reported access to HIV prevention programs specifically for sex workers and few reported participating in a research project related to sex work before. In addition, disclosure of sex work status to both health care workers and family was uncommon. There are providers of education and safe HIV testing services for sex workers in Swaziland, but these data suggest that coverage remains limited. Limited coverage is likely due to both suboptimal levels of the provision of services but also limited uptake of services because of fear related to inadvertent disclosure of sex work status. Criminalization of sex work across Africa including in Swaziland may impede FSW access to health services [30]. These results highlight the need to address both the quantity and quality of service provision by increasing the clinical and cultural competence in addressing the health care needs of sex workers in a rights-affirming manner [31], [32].

The interpretation of these data reported here appear to somewhat contrast with those of the 2009 Swaziland MoT study. To date, there were limited quantitative inputs for the MoT study including the size and density of sexual networks of FSW, the burden of HIV, sexual practices, and population size. Additionally, the existing MoT studies do not dynamically assess relative contribution of sex work given the limited ability to fully characterize chains of HIV transmission among these women and clusters of infection. Advanced methods of MoT such as those suggested by Mishra et al in this collection may better characterize the actual contribution of sex workers and their clients to generalized HIV epidemics.

There are several limitations to the methods used in this study. Causality of the associations with HIV cannot be firmly established using cross-sectional data. The use of RDS increases the external validity of the findings by adjusting for network size, homophily, and by limiting accrual by any one participant [15]. However, RDS is not probability sampling as there is no sampling frame from which to accrue participants, which limits the generalizability of findings [33]. To be conservative, we presented the crude and weighted analyses for prevalence estimates but used unweighted data for bivariate and multivariate modeling [34]. However, we also completed sensitivity analyses by comparing these results to those results obtained when using weighted results in the modeling and found little difference. Swaziland is geographically a small country, and seed participants were accrued in different regions of the country. However, the site was in a central location in the country, and the majority of the participants were from one of the two large urban centers in the country, which may overestimate the levels of service access and education levels for sex workers in the country.

## Conclusions

The 2011 Swaziland HIV Incidence Measurement Survey (SHIMS) demonstrated 54.5% HIV prevalence among women in Swaziland with equal to or greater than two sexual partners in the previous 6 months and 43.2% among those who were not married and ever had sex [35]. While our study only measured prevalence among FSW, the demographic characteristics of the highest risk women in the SHIMS survey matches that of the FSW sampled for this study. While there are not population size data available for FSW or their male clients in Swaziland, the speed and ease of accrual of 328 participants suggests that there is a sizable population of FSW in the country though this should be confirmed with appropriate methods. Moreover, the population attributable fraction of the total HIV epidemic in the country attributable to sex work has likely been underestimated. While Swaziland has a highly generalized HIV epidemic, reconceptualizing the needs of key populations such as FSW suggests that these women represent a distinct population with specific vulnerabilities and a high burden of HIV compared to other women. These women are understudied and underserved resulting in a limited characterization of their HIV prevention, treatment, and care needs and only sparse specific and competent programming. FSW are an important population for further investigation and rapid scale-up of combination HIV prevention including biomedical, behavioral, and structural interventions.
